# Raman Spectroscopy-Based Quality Control of “Silicon-On-Insulator” Nanowire Chips for the Detection of Brain Cancer-Associated MicroRNA in Plasma

**DOI:** 10.3390/s21041333

**Published:** 2021-02-13

**Authors:** Kristina A. Malsagova, Vladimir P. Popov, Igor N. Kupriyanov, Tatyana O. Pleshakova, Rafael A. Galiullin, Andrey F. Kozlov, Ivan D. Shumov, Dmitry I. Larionov, Fedor V. Tikhonenko, Svetlana I. Kapustina, Vadim S. Ziborov, Oleg F. Petrov, Olga A. Gadzhieva, Boris A. Bashiryan, Vadim N. Shimansky, Alexander I. Archakov, Yuri D. Ivanov

**Affiliations:** 1 Laboratory of Nanobiotechnology, Institute of Biomedical Chemistry, 119121 Moscow, Russia; t.pleshakova1@gmail.com (T.O.P.); rafael.anvarovich@gmail.com (R.A.G.); afkozlow@mail.ru (A.F.K.); shum230988@mail.ru (I.D.S.); corvus.coraxnm@gmail.com (D.I.L.); sveta.kapustina7.05@gmail.com (S.I.K.); ziborov.vs@yandex.ru (V.S.Z.); alexander.archakov@ibmc.msk.ru (A.I.A.); yurii.ivanov.nata@gmail.com (Y.D.I.); 2 Rzhanov Institute of Semiconductor Physics, Siberian Branch of Russian Academy of Sciences, 630090 Novosibirsk, Russia; popov@isp.nsc.ru (V.P.P.); ftikhonenko@gmail.com (F.V.T.); 3 Sobolev Institute of Geology and Mineralogy, Siberian Branch of Russian Academy of Sciences, 630090 Novosibirsk, Russia; spectra@igm.nsc.ru; 4 Joint Institute for High Temperatures of Russian Academy of Sciences, 125412 Moscow, Russia; ofpetrov@ihed.ras.ru; 5 Federal State Autonomous Institution “N. N. Burdenko National Medical Research Center of Neurosurgery” of the Ministry of Health of the Russian Federation, 125047 Moscow, Russia; ogadjieva@nsi.ru (O.A.G.); bbashiryan@nsi.ru (B.A.B.); Shimava@nsi.ru (V.N.S.)

**Keywords:** nanowire biosensor, sensor chip, silicon-on-insulator, brain cancer, diagnostics, Raman spectroscopy, micro RNA

## Abstract

Application of micro-Raman spectroscopy for the monitoring of quality of nanowire sensor chips fabrication has been demonstrated. Nanowire chips have been fabricated on the basis of «silicon-on-insulator» (SOI) structures (SOI-NW chips). The fabrication of SOI-NW chips was performed by optical litography with gas-phase etching. The so-fabricated SOI-NW chips are intended for highly sensitive detection of brain cancer biomarkers in humans. In our present study, two series of experiments have been conducted. In the first experimental series, detection of a synthetic DNA oligonucleotide (oDNA) analogue of brain cancer-associated microRNA miRNA-363 in purified buffer solution has been performed in order to demonstrate the high detection sensitivity. The second experimental series has been performed in order to reveal miRNA-363 itself in real human plasma samples. To provide detection biospecificity, the SOI-NW chip surface was modified by covalent immobilization of probe oligonucleotides (oDNA probes) complementary to the target biomolecules. Using the SOI-NW sensor chips proposed herein, the concentration detection limit of the target biomolecules at the level of 3.3 × 10^−17^ M has been demonstrated. Thus, the approach employing the SOI-NW chips proposed herein represents an attractive tool in biomedical practice, aimed at the early revelation of oncological diseases in humans.

## 1. Introduction

Raman spectroscopy is based on an inelastic photon scattering, also known as the Raman effect, discovered by K.V. Raman in 1928. It represents a non-invasive optical method, which can be employed as a supplementary method in the diagnosis of certain diseases [1], and as an alternative to other more invasive methods, such as biopsy [2]. Moreover, Raman spectroscopy finds its application in various forms of research aiming at the development of biosensors [3,4]. So, in [5], the use of a microfluidic chip based on surface-enhanced Raman spectroscopy (SERS) was described, and applications of SERS-based substrates in microfluidic technology was discussed. The substrates described therein [5] provide the development of microfluidic SERS-based chips, which allow one to detect target molecules in real time. This is quite useful for biomedical studies. Such SERS-based chips, however, do not provide sufficiently high detection sensitivity (with lower limits of detection of the orders of 10^−17^ to 10^−15^ M) required for early diagnosis of cancer [6]. Nevertheless, Raman spectroscopy can be successfully employed for the quality control in systems with higher sensitivity. In this way, the high sensitivity is only required from the device directly used for the registration of target molecules in molecular detection mode. The proper operation of such a molecular detector is achieved owing to high quality of its fabrication. For instance, in the case of chips for atomic force microscopy (AFM), the chip surface must be atomically smooth, and the surface roughness of the chip is controlled by AFM. 

Moreover, high quality required from SERS substrates still represents the factor limiting wide application of SERS. However, at present—unlike the situation observed two decades ago—the most attention is paid to the development of SERS-based substrates with low fluctuation of the signal, low fluorescence (or photoluminescence) level, high homogeneity and stability, high affinity to the target and low number of desorption and photoinduced reactions [7].

In developed countries such as the UK, brain cancer accounts for approximately 2% of all cases of cancer in adults. It should be, however, emphasized that worldwide, threefold differences in the occurrence of brain tumours have been reported between various countries. Namely, highest rates of brain tumours appear to be observed in developed countries. This, however, can be owing to better registration systems, which contain benign tumours [8]. The greatest proportion of tumours in adults are supratentorial ones, which arise in the frontal, temporal and parietal lobes. The majority (86%) of these tumours are gliomas, which include astrocytomas, glioblastomas, oligodendroblastomas, and unspecified gliomas [8]. Despite a considerable improvement during the last decades, survival rate of brain cancer patients remains poor [8,9].

To date, there is no standard diagnostic or therapeutic strategies for the early diagnosis of brain cancer, and this is why the early revelation of brain cancer remains to be a complex task. The future development of such methods, as liquid biopsy [10] and serological detection of biomarkers [11] can, however, allow for the early diagnosis and timely treatment of brain cancer and, accordingly, can improve the disease prognosis and survival chances. With respect to serological diagnostics, there is a problem related to insufficient sensitivity of detection of the cancer markers. That is, diagnosis of cancer at an early stage, when the concentration of marker biomolecules in the serum is low, requires the development of highly sensitive methods of cancer marker detection, which allow one to attain, at least, a femtomolar (~10^−15^ M) detection limit [6,12]. In this regard, the application of Raman spectroscopy in the development of highly sensitive nanotechnology-based diagnostic devices is promising.

With respect to cancerogenesis, many mechanisms can regulate the development of cancer. In this way, during recent decades, non-coding ribonucleic acids (RNAs) were, gradually, recognized to participate in the development of cancer [13]. In contrast to protein-encoding genes, non-coding RNAs can regulate gene expression in the course of or after the transcription, as well as during the translation. Recently, microRNAs (miRNAs), whose features are partially similar to those of non-coding RNAs, were identified to be cancer markers [14,15,16]. Accordingly, the use of miRNAs as serologic markers of cancer is promising [17,18].

The exact role of cancer-specific miRNAs is still incompletely studied [19,20,21,22,23,24]. MiRNAs reversibly regulate transcription through nonmutation mechanisms, and can thus be considered as epigenetic effectors [25]. Among miRNAs, associated with brain cancer, one should single-out miRNA-363, whose upregulation is supposed to play a role in malignant glioma signature [26].

To date, no “gold standard” method for quantitative analysis of miRNAs was developed. Direct miRNA hybridization on DNA microchips and real-time polymerase chain reaction (PCR) are currently most often used for quantitative analysis of miRNAs. The DNA microchip-based method is, however, insufficiently sensitive, and requires relatively large quantities of sample material, while its dynamic range is ~3 orders of magnitude [27]. In contrast, the dynamic range of real-time PCR makes up seven orders of magnitude, while the method itself is characterized by high sensitivity and specificity, and small quantities of sample material are required [28]. The disadvantages of real-time PCR consist in the use of reverse transcription (whose efficiency can be low) and high sensitivity to sample contamination. The latter often leads to obtaining false-positive results, since the amplification of amino acid molecules is performed in PCR.

Next-generation sequencing (NGS) allows one to obtain miRNA expression profiles [29] and to detect both known and unidentified miRNAs. However, since this method is connected with producing large datasets, the detection and identification of miRNAs represents a complex task, which requires bioinformatic resources and development of specialized algorithms [30]. Along with complicated data analysis, NGS is characterized by high cost and large amounts of sample required. In addition, the absolute amount of miRNA cannot be determined by NGS [27,31]. For this reason, the development of novel methods for the early revelation of cancer represents an urgent problem of modern biomedical research.

In this way, one of the promising directions in modern biomedical research is the development of molecular detectors with ultra-high sensitivity. These devices allow one to detect single biological macromolecules. In addition, the use of such detectors does not require amplification. That is, molecular detectors are devoid of the disadvantages mentioned above, which are characteristic for conventional detection systems.

Once again, molecular detectors allow one to count single target macromolecules. Accordingly, upon using such detectors, the amplification of target molecules is not required for their detection at ultra-low concentrations. Nanowire (NW) biosensors represent one of the types of molecular detectors. Their use for the highly sensitive detection of various biomolecules [32,33,34] and viral particles [35] was previously demonstrated in a number of papers. These biosensors allow for label-free detection of biological macromolecules in real time with high sensitivity (at femtomolar and even subfemtomolar level). These advantages allow nanowire biosensors to be considered as a basis for the development of novel analytical systems intended for biomedical applications.

The principle of operation of a NW biosensor is based on the registration of a modulation of an electric current, flowing through the NW sensor structure upon adsorption of analyte molecules onto its surface [36,37,38,39]. The high surface-to-volume ratio and the one-dimensional nanoscale morphology of the sensor structures promote fast diffusion of the target molecules both towards and from the sensor surface, and allow for high sensitivity of the NW biosensor. Accordingly, this allows one to increase the reaction rate and, hence, leads to higher sensitivity and faster response and recovery time. This is why the development of silicon-on-insulator nanowire (SOI-NW) chips with smallest dimensions of sensor elements represents a relevant task. Theoretical detection limit, attainable with using a NW biosensor, can reach the level of single molecules [40]. To date, however, this is connected with a problem, consisting in proper quality control of such sensor chips: the presence of defects and stresses significantly impair the current characteristics of the NW biosensor sensor elements. To overcome this problem, we have developed a procedure for monitoring the quality of fabrication of such sensor chips by Raman spectroscopy.

In the present study, micro-Raman spectroscopy has been employed for monitoring the formation processes of the SOI-NW chip surface. The good quality of the fabricated SOI-NW sensor chips has been demonstrated. This has allowed to use these chips in the experiments on the highly sensitive detection of brain cancer-associated miRNA. Namely, two series of such experiments have been conducted. In the first experimental series, detection of a synthetic DNA oligonucleotide (oDNA) analogue of brain cancer-associated microRNA miRNA-363 in purified buffer solution has been performed in order to demonstrate the high (3.3 × 10^−17^ M) detection sensitivity. The second experimental series has been performed in order to re-veal miRNA-363 itself in real human plasma samples. To provide the analysis biospecificity, the NW sensor surface was sensitized by covalent immobilization of DNA oligonucleotide (oDNA) probes, which are known to be complimentary to the sequence of the target molecules. In the experiments with the detection of synthetic oDNA in buffer, it has been demonstrated that our SOI-NW chips with immobilized oDNA probes can be successfully employed for the detection of their complementary oDNAs with a very high, 3.3 × 10^−17^ M concentration sensitivity. In the experiments with plasma samples, the successful application of the SOI-NW chips, developed herein, for the detection of brain cancer-associated miRNA-363 has been demonstrated.

## 2. Materials and Methods

### 2.1. Chemicals

The following chemicals were used throughout our experiments: potassium phosphate monobasic (KH_2_PO_4_, Sigma-Aldrich, St. Louis, MO, USA); isopropanol (99.9% pure, Acros Organics, Geel, Belgium); hydrofluoric acid (HF, Reakhim, Moscow, Russia); ethanol (C_2_H_5_OH, 96% pure, Reakhim); 3-aminopropyltriethoxysilane (APTES, Sigma-Aldrich); 3,3′-dithiobis (sulfosuccinimidyl propionate) (DTSSP, Pierce, Waltham, MA, USA). Deionized water was obtained using a Simplicity UV system (Millipore, Molsheim, France).

### 2.2. Oligonucleotides

For the sensitization of the surface of working sensors, we used the oDNA probe with the following sequence: 5′-NH2-(T)_10_-GGTTTACAGATGGATACCGTGCAATTTTTCT CCTATGATACTCATCAAAATTGCATCGTGATCCACCCGACAACA; this sequence is complementary to that of the target oDNA (TGTTGTCGGGTGGATCACGATGCAATTTTGATGAGTATCATAGGAGAAAAATTGCATATCCATCTGTAAACC), which corresponds to that of the target microRNA-363 (Evrogen, Moscow, Russia).

### 2.3. Plasma Samples

The patients were examined in the National Medical Research Center for Neurosurgery named after Academician N. N. Burdenko of the Russian Federation (Moscow, Russia). In our experiments, control plasma samples of healthy volunteers, and plasma sample no. 001 of a patient suffering from anaplastic oligodendroglioma (Grade III) were tested. All experiments involving the plasma were performed in compliance with the Order no. 1177n (Ministry of Health of Russian Federation, 20 December 2012). Plasma samples were obtained from patients according to the patient examination protocol. This study was approved by independent ethical committee organized on the basis of the National Medical Research Center for Neurosurgery named after Academician N. N. Burdenko of the Russian Federation. Written informed consent was obtained from the patient and from healthy volunteers, authorizing their participation in the study and the use of the biological material. To provide biological safety, all the samples were deactivated prior to their use in the study.

For the isolation of miRNAs from the blood plasma samples, the miRCURY^™^ RNA Isolation Kit—Biofluids (Exiqon A/S, Vedbaek, Denmark) was employed; the isolation was performed according to the protocol provided by the manufacturer.

### 2.4. Fabrication of the SOI-NW Chips

The chips were fabricated by nanostructuring, employing SOI-NW structures of n-type conductance. The characteristics of the NW sensors were as follows: the cut-off silicon layer thickness was 32 nm; the buried oxide (BOX) layer thickness was 300 nm; the width, the thickness and the length of each nanowire sensor were 3 µm, 32 nm and 10 µm, respectively. The contact of aluminium with n-type silicon represents a Schottky barrier with a height of up to 0.3 V. 

To obtain the micro-Raman spectra, a Horiba Jobin-Yvon LabRam HR800 Raman spectrometer (HORIBA, Kyoto, Japan) was employed. The presence of a built-in Olympus BX41 microscope (Olympus Corp., Tokyo, Japan; objective focal distance 1 mm; N.A. = 0.65; analyzed area diameter < 3 µm) allowed us to investigate sub-micrometer areas of the channels of field-effect transistors (FETs) with the coating. A confocal optical scheme allowed us to achieve maximum degree of detail (which is necessary due to small dimensions of the sensor elements), while maintaining a high speed of image acquisition upon excitation by a focused beam of argon or fiber-optic laser (514 nm, 0.1 to 1.0 mW) to prevent heating of the coated nanosensor surface. The confocal scheme also allowed us to measure the properties of the dielectric layer directly on silicon. 514 nm laser wavelength allowed us to observe dispersion of Raman peaks, as well as to separate them from luminescence peaks. 

### 2.5. Surface Modification of SOI-NW Sensor Chips 

To remove organic contaminants, the surface of the SOI-NW sensor chips was treated, as described elsewhere [32,41,42], with aqueous isopropanol solution, and then with a solution containing HF and ethanol (in order to remove the native oxide layer formed during the chip storage). After such a treatment, the chip was incubated in an ozonator (UV Ozone Cleaner–ProCleaner^™^ Plus, BioForce Nanosciences, (Ossila Ltd., Sheffield, UK) in order to form hydroxyl groups on the SOI-NW surface, then it was silanized with APTES (as described in our previous papers [41,43,44]), washed with ethanol and dried [41].

### 2.6. Covalent Immobilization of oDNA Probes 

In order to achieve biospecific interaction of the SOI-NW sensor surface with target oDNA/miRNA biomolecules, the surface of the NW sensors was sensitized with covalently immobilized oDNA probes, whose oligonucleotide sequence was complimentary to that of the target biomolecules. This procedure was performed similarly to that described in [41]. Namely, the immobilization was performed with using a preliminary activation of the chip surface with DTSSP crosslinker, and then ~0.6 nL microdrops of oDNA probes’ solutions (1 µM in 50 mM potassium phosphate buffer) were precisely dispensed onto the activated surface of individual SOI-NWs sensors with a Piezorray system (Perkin Elmer, Inc., Waltham, MA, USA). The oDNA solutions were incubated on the activated SOI-NW surface at 15 °C for 0.5 h, and then washed away.

### 2.7. Preparation of Solutions of Target oDNA in Buffer

Solutions of target oDNA were prepared from the initial 100 µM stock solution in 50 mM potassium phosphate buffer (pH 7.4) by serial tenfold dilution with working buffer (1 mM potassium phosphate buffer, pH 7.4) analogously to [41] and used in biosensor experiments immediately after preparation.

### 2.8. Electrical Measurements

Electrical measurements were performed employing a ten-channel data acquisition and storage system (“Agama+” JSC, Moscow, Russia) similar to [41]. Time dependencies of the drain-source current *I_ds_*(*t*) were recorded in real time at *V_g_* = 40 V and *V_ds_* = 0.1 V [32,41,44].

### 2.9. Biosensor Measurements

The biosensor system used in our experiments was similar to [41], comprising an analytical unit and an electronic measuring unit. The description of the biosensor system was reported in detail in our previous paper [41].

For the detection of target oDNA in buffer, the oDNA solution of concentration ranging from 1.0 × 10^−16^ M to 1.0 × 10^−14^ M (150 μL in 1 mM potassium phosphate buffer) was added into the measuring cell containing 300 μL of pure buffer. Control experiments were performed under the same conditions, but pure oDNA/miRNA-free buffer solution was added into the cell.

For the detection of miRNA in plasma, 7 µL of the sample, containing miRNA isolated from the analyzed plasma sample as described in Section 2.3, were added into the measuring cell containing 100 μL of pure buffer. Control experiments were performed under the same conditions, but the sample of a healthy volunteer was added into the cell instead of the sample of a patient. To avoid the Debye screening effect, the biosensor measurements were carried out in a low-salt buffer (1 mM potassium phosphate buffer) [45].

The biosensor response current signal was recorded in real time, and the signal in relative units was calculated analogously to the technique described elsewhere [32]. The so-obtained time dependencies of the biosensor signal were presented in the form of sensogram curves. 

## 3. Results

### 3.1. Monitoring of Quality of SOI-NW Chips by Micro-Raman Spectroscopy

**Figure 1:**
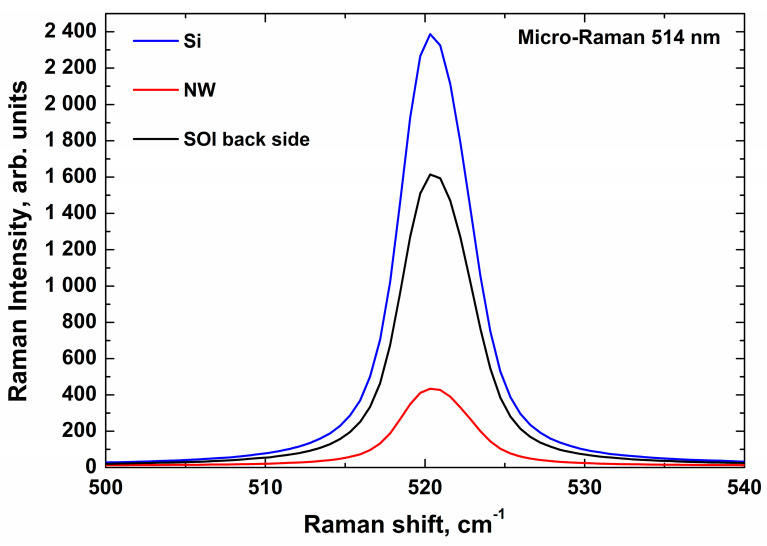
Typical micro-Raman spectra obtained using a 514-nm-wavelength laser beam, which was directed perpendicularly to the NW surface along the silicon (100) axis. Si—control silicon crystal; NW—nanowire; SOI back side—silicon substrate of the SOI structure.

Figure 1 displays typical micro-Raman spectra illustrating the monitoring of quality of SOI-NW chips by micro-Raman spectroscopy. The spectra were excited by laser radiation in visible range at 514 nm. Typical spectra, obtained for a silicon crystal (Si), and for both the substrate (SOI back side) and the nanowire (NW) of a SOI structure. Silicon crystal was used as a control defect-free, stress-free structure. Raman spectra of silicon have been studied in order to compare the characteristics of the silicon nanowire and the substrate of the SOI structure with those of silicon.

As one can see from Figure 1, the typical Raman spectra, obtained for both the nanowire and the substrate of the SOI structure, and that obtained for the control silicon crystal, have their maxima at one and the same wavelength. This indicates that the structure of the silicon nanowire is virtually free from stresses and defects. The latter indicates a good quality of the fabricated nanowire structures and, hence, allows one to use a chip with these structures in biosensor experiments. Below, the results of experiments with nanowire chips, containing these sensor structures, are presented.

**Figure 2:**
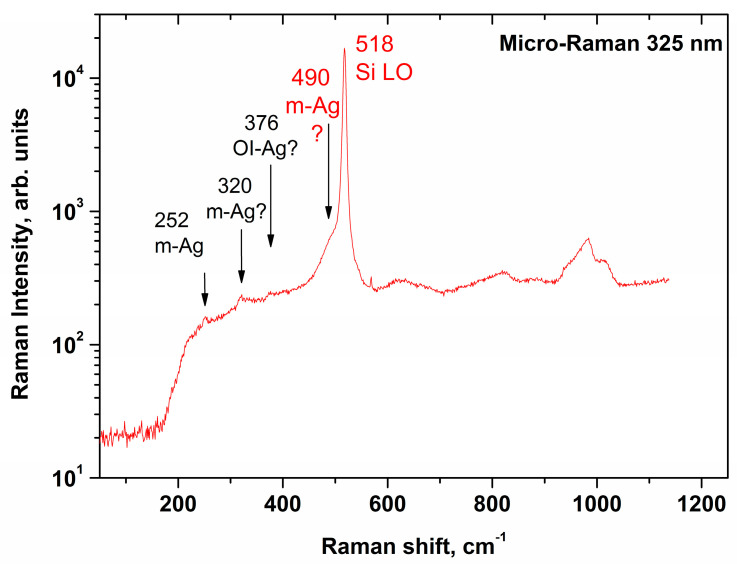
Micro-Raman spectrum, acquired for SOI structure in the direction of the UV laser beam along the silicon surface and along the (110) axis after annealing at 1100 °C and a cycle of measurements.

As an example of monitoring stretching effects in SOI structures, below, we present Raman spectrum of a structure with a Al_2_O_3_/HfO_2_ coating. The spectrum was excited by laser radiation in UV range (at 325 nm). Figure 2 displays an example of the so-obtained micro-Raman spectrum.

The UV-excited micro-Raman spectrum obtained for the SOI structure, shown in Figure 2, indicates that the signal of single-phonon scattering on LO phonons of silicon (520.5 cm^−1^) is shifted towards lower wavenumbers. This probably indicates the presence of tensile stresses due to defects at the oxide interface, or due to layer-by-layer oxidation defects [46]. However, further studies are required to unambiguously answer this question.

### 3.2. Detection of oDNA and miRNA-363, Associated with Brain Cancer

In the first step, the functionalization of the sensor surface was performed. The functionalization comprised of two steps: (1) chemical modification of the sensor surface, and (2) its sensitization with oligonucleotide molecular probes.

The modification of the chip surface was carried out to obtain an organic silane layer with terminal primary amine groups exposed. This procedure is necessary for further covalent attachment of the molecular probes to the sensor surface. After that, accordingly, the molecular probes, complimentary to the target oDNAs (which represented synthetic analogues of miRNA-363), were covalently immobilized onto the sensor surface as described in Materials and Methods. Afterwards, the biosensor detection of the target molecules was performed.

**Figure 3:**
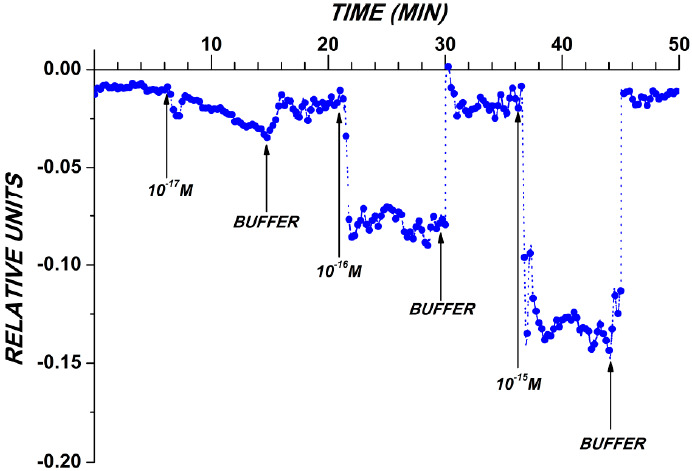
The results obtained in the course of the detection of target oDNA in the buffer with the use of a SOI-NW chips with n-type conductance, sensitized with covalently immobilized oDNA probes. Typical sensogram curves obtained upon the analysis of solutions with various oDNA concentrations. Experimental conditions: the concentration of target oDNA in the measuring cell was 3.3 × 10^−17^ M; 3.3 × 10^−16^ M; 3.3 × 10^−15^ M; 1 mM potassium phosphate buffer (pH 7.4); *V_g_* = +40 V; *V_ds_* = 0.1 V. The total solution volume in the cell was 450 µL. Arrows indicate the addition of target oDNA solution and the wash with a pure potassium phosphate buffer.

Two series of experiments on the detection of target molecules were conducted. In the first series, detection of a synthetic DNA oligonucleotide (oDNA) analogue of brain cancer-associated microRNA (miRNA-363) in purified buffer solution at concentrations of the target oDNA from 3.3 × 10^−17^ M to 3.3 × 10^−15^ M was performed in order to demonstrate the high detection sensitivity. Figure 3 displays typical sensogram curves obtained in this series. The oDNA solution was added into the measuring cell containing working buffer. To account for the non-specific adsorption, an additional control NW sensor, located on the same chip, was employed. The surface of the control NW sensor was free from immobilized oDNA.

The data shown in Figure 3 indicate a decrease in the biosensor signal level with decreasing the target oDNA concentration from 3.3 × 10^−15^ to 3.3 × 10^−17^ M was observed in these conditions. In the control experiments, when the oDNA-free buffer was added into the measuring cell, either no signal from the nanowire sensors was registered, or the change in the level of the biosensor signal amounted to only 1% to 2% from the baseline level of the current (data not shown).

The sensogram curves obtained in this experimental series also indicate that the signal returns to the baseline level after the oDNA solution in the measuring cell is replaced with pure oDNA-free buffer. These results indicate that the composition of the washing buffer developed provides efficient regeneration of the sensor surface.

**Figure 4:**
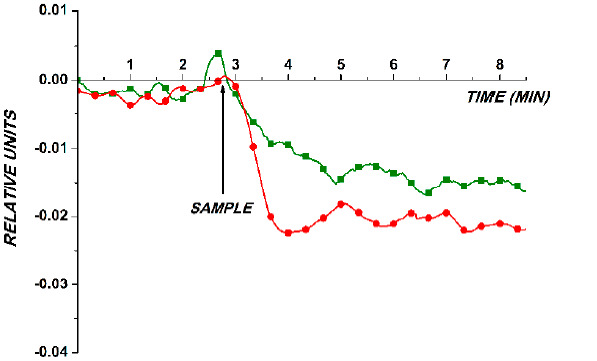
Typical sensogram curves obtained with the use of the oDNA-senstized SOI-NW chip with n-type conductance, obtained upon the analysis of 3.3 × 10−17 M solution of a synthetic oDNA analogue of miRNA-363 in purified buffer (green line and squares ■), and upon the analysis of microRNA isolated from plasma of a brain cancer patient (red line and circles ●). Experimental conditions: 1 mM potassium phosphate buffer, pH 7.4; *V_g_* = +40 V; *V_ds_* = 0.1 V. Arrows indicate the sample addition and the washing with pure potassium phosphate buffer.

The second experimental series has been performed in order to reveal miRNA-363 itself in real human plasma samples. The sample, containing miRNA isolated from the analyzed plasma sample as described in Section 2.3, was added into the measuring cell containing pure buffer. Control experiments were performed under the same conditions, but the sample of a healthy volunteer was added into the cell instead of the sample of a patient. The results obtained in the course of the detection of target molecules in purified buffer and in the sample of a patient are shown in Figure 4. The signal level obtained in control experiments was negligible, and these data are not shown in order to avoid cluttering in the figure.

The curves shown in Figure 4 demonstrate a decrease in the biosensor signal level after the addition of both the purified 3.3 × 10^−17^ M solution of target oDNA and the sample of a brain cancer patient. In the control experiments, after either the oDNA-free buffer or the sample of a healthy volunteer was added into the measuring cell, the change in the biosensor signal level was negligible. This indicates a biospecific interaction between the sensor-immobilized oDNA probes and the target oDNA/miRNA-363 molecules, captured onto the sensor surface from the analyzed solution. The comparison of the curves shown in Figure 4 indicates that the concentration of the target molecules in the purified oDNA solution and that in the plasma sample are approximately of the same order of magnitude (10^−17^ M).

## 4. Discussion

Herein, we have employed Raman spectroscopy in order to monitor the quality of SOI-NW chips. In the spectroscopic experiments, laser radiation of the visible range was employed. The laser beam was directed perpendicularly to the NW surface along the silicon (100) axis. In the spectrum, obtained for the nanowire and the substrate (SOI back side) fabricated, a characteristic Raman shift was observed, and the position of its maximum (peak) coincided with that obtained for the pure silicon. Such an approach allowed us to rapidly monitor the quality of the NW sensors fabrication. Namely, the same position of the peak and its shape indicate that the structure of the silicon nanowire fabricated is free from any stresses and defects. Such a good quality of the nanowire allows one to use it for the highly sensitive detection of biological macromolecules—such as miRNAs, which can be used as promising biomarkers of brain cancer in humans. The quality monitoring is quite necessary, since poor quality of the NW, consisting in the presence of stresses and defects in it, impedes the detection sensitivity.

NW structures are now actively used in the development of novel methods for diagnostics. At the same time, miRNA are now considered as new promising markers of various diseases. In this way, the scientific group by T. Yasui has proposed a device, consisting of nanowires fixed on a microfluidic substrate [47]. Such a device allows one to collect extracellular vesicles with high efficiency, and to extract in situ about 1000 miRNA types with different sequences. The number of extractable miRNA types obtained by Yasui et al. is significantly greater than that attainable by conventional ultracentrifugation. Moreover, the technique proposed by Yasui et al. can be employed for the detection of miRNAs in urine. These miRNAs can represent potential markers of not only bladder and prostate cancer, but also of non-urological cancer (lung, pancreas and liver cancer). Such an approach also allowed the authors to detect about 20 miRNAs in urine, but these miRNAs were not identified as candidate markers of cancer. Accordingly, the approach proposed by Yasui et al. requires further investigation to determine its specificity.

In the paper by Fan et al. [48], an array of biosensors, based on microelectrodes with nanometer-size gate has been fabricated. These authors immobilized molecular probes in nanogaps of a pair of microelectrodes, with following hybridization with target miRNAs. Fan et al. demonstrated that under optimal conditions, target miRNA can be analyzed quantitatively at concentrations ranging from 10 fM (1 × 10^−14^ M) to 20 pM (2 × 10^−11^ M). The authors showed that such an array of biosensors can be used for direct detection of target miRNA in the mixture of miRNAs extracted from cancer cell lines.

The NW structures, fabricated in our study, have further been used for the highly sensitive detection of a synthetic oDNA analogue of brain cancer-associated miRNA-363. Namely, the SOI-NW sensor chips were employed for the rapid detection of the target oDNA at concentrations of the order of 3.3 × 10^−17^ M—that is, within the subfemtomolar range. This concentration range is of particular interest for the early revelation of oncological diseases in humans [6]. The revelation of brain cancer at an early stage is particularly important, since this disease is characterized by a poor survival rate [8,9].

In our previous papers, we also demonstrated that the (nanowire biosensor)-based approach can be successfully employed for the detection of protein [32,43,44,49] and nucleic acid [41,42,50] biomolecules in plasma samples. Herein, the SOI-NW sensor chips with high-quality sensor chips has been demonstrated to be suitable for the highly sensitive detection (3.3 × 10^−17^ M) of relatively small nucleic acid molecules in both purified buffer solutions and plasma samples. Thus, the approach proposed herein, which includes Raman spectroscopy-based rapid monitoring of the sensor fabrication quality, can be well used as the basis for the development of novel biomedical devices, intended for the early revelation of cancer (as well as other diseases), in clinical practice.

## 5. Conclusions

Herein, micro-Raman spectroscopy has been employed for the quality monitoring of nanowire sensor chips’ fabrication. The nanowire chip represented a «silicon-on-insulator» (SOI) structure. To obtain the Raman spectra of the nanowire sensors, a laser radiation of visible range was used. The spectra obtained indicated a good quality of the fabricated nanowires. This allowed us to use the nanowires for the highly sensitive detection of brain cancer-associated miRNA-363 molecules. With using a purified solution of a synthetic oDNA analogue of miRNA-363 in buffer, the ultra-high (3.3 × 10^−17^ M) concentration sensitivity of the SOI-NW biosensor has been demonstrated. In our experiments, the successful detection of miRNA-363 in human plasma has also been demonstrated. The approach proposed herein can further be used in the development of novel highly sensitive biosensor-based methods, intended for biomedical applications.

## Data Availability

Correspondence and requests for materials should be addressed to Y.D.I.

## Abbreviations

AFM	atomic force microscopy
DNA	deoxyribnucleic acid
DTSSP	3,3′-dithiobis (sulfosuccinimidyl propionate)
FET	field-effect transistor
miRNA	micro-(ribonucleic acid)
NGS	Next-generation sequencing
NW	nanowire
oDNA	deoxyribnucleic acid oligonucleotide
PCR	polymerase chain reaction
RNA	ribonucleic acid
SERS	surface-enhanced Raman spectroscopy
SOI	«silicon-on-insulator»
SOI-NW chips	nanowire chips based on «silicon-on-insulator» structures
UV	ultraviolet
